# The Effect of Aligning Childhood Influenza Vaccination with Specific Well-Visits in a Primary Care Institution in Singapore

**DOI:** 10.3390/vaccines14060469

**Published:** 2026-05-25

**Authors:** Ziying Goh, Yi Ling Eileen Koh, Wai Keong Aau, Ngiap Chuan Tan, Chirk Jenn Ng, Chung Wai Mark Ng

**Affiliations:** 1 SingHealth Polyclinics, Singapore 150167, Singapore; eileen.koh.y.l@singhealth.com.sg (Y.L.E.K.); aau.wai.keong@singhealth.com.sg (W.K.A.); tan.ngiap.chuan@singhealth.com.sg (N.C.T.); ng.chirk.jenn@singhealth.com.sg (C.J.N.); ng.chung.wai@singhealth.com.sg (C.W.M.N.); 2 Centre for Population Health Research and Implementation, SingHealth Regional Health System, Singapore 150167, Singapore

**Keywords:** influenza vaccination, childhood vaccination, children, well-visits, primary care

## Abstract

**Background/Objectives**: Influenza vaccination in young children reduces the incidence of influenza-related complications. Compared to other vaccines in the Singapore National Childhood Immunization Schedule (NCIS), there is low influenza vaccine uptake rate among young children. Our retrospective database study aimed to evaluate the effect of aligning childhood influenza vaccination to coincide with specific well-visits in the primary care setting. **Methods**: A retrospective interrupted time series study was conducted on two cohorts of children aged 6 to 12 months (n = 10,082 before, and n = 9234 after). The delivery schedule was aligned with routine touchpoints to administer Dose 1 of the influenza vaccine at the 6-month well-visit and Dose 2 at the 7-month visit. **Results**: Dose 1 influenza vaccination rates increased from 4.7% before the intervention to 73.7% after the intervention. The proportion of children who completed two doses of the influenza vaccines increased from 3.6% to 62%. Median age at Dose 1 influenza vaccine, and completion of two doses of influenza vaccine decreased from 8.7 to 7.6 months and 9.9 to 7.7 months, respectively. **Conclusions**: Aligning influenza vaccination with specific well-visits substantially improves uptake, completion rates, and timeliness of vaccination, demonstrating a scalable system-level strategy to enhance immunization coverage in primary care.

## 1. Introduction

Seasonal influenza virus is a common cause of lower respiratory tract infection (LRTI) with significant morbidity and mortality in young children. In 2018, among children under 5 years globally, there were an estimated 10.1 million influenza-virus-associated LRTI cases resulting in 870,000 hospital admissions and 34,800 deaths. Influenza virus also accounted for 7% of LRTI cases, 5% of LRTI hospital admissions, and 4% of LRTI deaths in children under 5 years [1].

In tropical Singapore, influenza infection happens throughout the year. A study from the largest pediatric hospital in Singapore showed that the highest burden of influenza hospitalizations was in children younger than 6 months of age, followed by children between 6 months and 59 months of age [2]; majority (75.2%) of the children admitted had no comorbidity. The mortality rate in the study cohort was 0.2%, and children who were between 6 months and 59 months of age had the highest influenza-associated complication rate (30.6%). Neurological complications were the most frequent, comprising febrile seizures, status epilepticus, encephalitis, encephalopathy, aseptic meningitis, and myelitis in decreasing frequency. In another study, the number of children with febrile seizures presenting to a pediatric emergency department was significantly associated with influenza epidemic activity [3], and those with neurological complications had a higher mortality rate [2,4,5,6].

Influenza vaccination has been shown to reduce the disease burden [7]. The World Health Organization (WHO) recommends annual influenza vaccination in groups at risk of severe influenza or its complications [8]; these high-risk groups include young children aged between 6 months and 59 months. Children aged 6 months through 8 years who are receiving the influenza vaccine for the first time require two doses spaced at least 4 weeks apart to be fully protected [9,10]. In Singapore, the Ministry of Health (MOH) recommends annual influenza vaccination for children from 6 months to 59 months of age through the National Childhood Immunization Schedule (NCIS) [11]. All vaccines under NCIS are fully funded at public healthcare institutions for children who are Singapore citizens or Permanent Residents.

Despite being recommended and fully funded through NCIS since November 2020, uptake of influenza vaccination remains low. In 2023, a local study published reported that among preschool children aged 2 to 6 years only 27.5% had received influenza vaccination at some point in the past, with 11.7% having received influenza vaccine in the last 12 months [12]. In contrast, the other vaccines in the NCIS have a significantly higher uptake [13]. In 2020, the vaccine primary course coverage for children at 2 years of age for the NCIS vaccines were high: Bacillus Calmette–Guérin (BCG) at 98.5%, hepatitis B at 97.5%, diphtheria, pertussis, and tetanus at 97.9%, poliomyelitis at 97.9%, Hemophilus influenzae b (HIb) at 97.9%, pneumococcal at 91.4%, and measles, mumps, and rubella at 94.0%.

The low influenza vaccine uptake may be due to the lack of a specific well-visit touchpoint where children attend for developmental assessment and vaccinations for its recommendation. Unlike the other vaccines whose administration are scheduled to coincide with touchpoints during well-visits at specific ages, influenza vaccine is not tied to any specific well-visit. Instead, the national schedule recommends an age range during which influenza vaccine can be administered. In addition, parental and provider concerns of multiple injections at each well-visit poses a significant barrier [14,15,16].

Currently, there is limited research on the potential synergy of coupling influenza vaccine with specific well-visit touchpoints in childhood vaccination schedules [17,18,19,20]. As such, the primary objective of this study is to determine the effects of synchronizing childhood influenza vaccinations with specific well-visit touchpoints on vaccine uptake rate within a primary care setting.

## 2. Materials and Methods

### 2.1. Implementation of a Visit-Aligned Schedule

**Figure 1:**
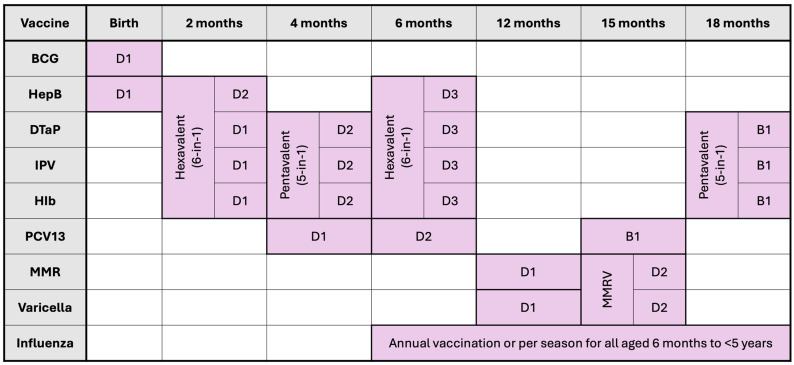
Pre-intervention childhood vaccination schedule [based on Singapore National Childhood Immunization Schedule (NCIS), 2020. From birth to 18 months. BCG = Bacillus Calmette–Guérin, HepB = Hepatitis B, DTaP = Diphtheria, tetanus and acellular pertussis, IPV = injectable polio vaccine, PCV13 = 13-valent pneumococcal conjugate vaccine, MMR = measles, mumps, and rubella, MMRV = measles, mumps, rubella, and varicella, D1, D2, and D3 = Doses 1, 2, and 3, respectively, B1 = Booster 1.

Prior to the intervention, we operationalized the National Childhood Immunization Schedule (NCIS) using the schedule illustrated in Figure 1. To minimize injection burden at each visit, the number of injections was limited to two per visit. When more than one injectable vaccine was administered at a single visit, injections were given at anatomically separate sites, typically in different limbs. In situations where more than one injection was required in the same limb, injection sites were spaced at least 2.5 cm apart to facilitate differentiation of local reactions, in accordance with recommended practice. Annual influenza vaccine was recommended as per national schedule without any alignment to any specific well-visit. In Singapore, inactivated influenza vaccines routinely supplied in public primary care institutions are administered via the intramuscular route. Notably, the NCIS also recommends a 3-dose childhood primary series for pneumococcal conjugate vaccine instead of a 4-dose series recommended in many other countries [11,17,18,19,20].

**Figure 2:**
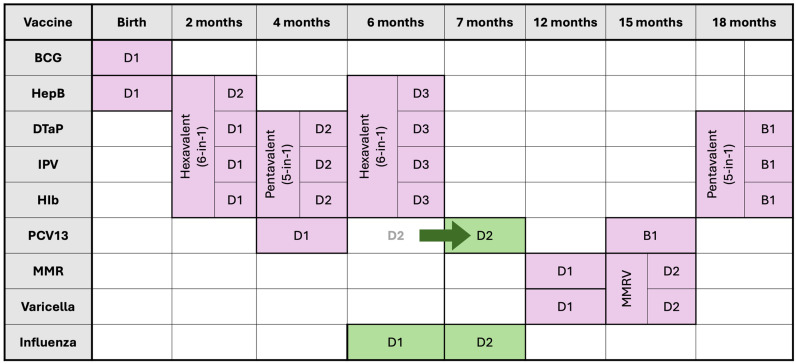
The visit-aligned schedule to operationalize the NCIS by anchoring Doses 1 and 2 of influenza vaccine at ages 6 and 7 months, respectively, and shifting Dose 2 of PCV13 vaccine to age 7 months (as depicted by the green arrow) to limit the number of injections to two per visit.

A visit-aligned schedule was introduced on 15 March 2023, anchoring Influenza Vaccine Dose 1 at the 6-month well-visit and Dose 2 at the 7-month visit (Figure 2). This approach was designed to translate a flexible age-based recommendation into defined clinical touchpoints, ensuring timely initiation and completion of the influenza vaccination series within the routine care pathway.

Implementation of the schedule required balancing clinical requirements with operational feasibility. Influenza vaccine-naïve children require two doses administered at least four weeks apart for optimal immunogenicity [9,10]. Therefore, a dedicated 7-month visit was established to facilitate timely completion of the primary series.

To limit the number of injections at two per visit, Dose 2 of the 13-valent pneumococcal conjugate vaccine (PCV13) was rescheduled from the 6-month visit to the 7-month visit and co-administered with the second dose of the influenza vaccine. The design of the schedule was informed by clinical consensus and discussed with senior subject-matter experts, including a Pediatric Infectious Diseases specialist involved in national immunization policy development. This adjustment was considered clinically acceptable based on the local epidemiology of invasive pneumococcal disease and ensured that the total number of injections per visit remained unchanged [21].

Prior to the implementation, engagement sessions were conducted with healthcare providers, including doctors and nurses, to introduce the revised schedule and address potential concerns. Standardized communication strategies were developed to support discussions with parents regarding the updated vaccination schedule. In addition, a contingency protocol was established to manage potential influenza vaccine supply interruptions. In the event of a stockout, clinics reverted to the standard flexible administration workflow, with influenza vaccination deferred until supply was restored, ensuring that other NCIS vaccinations were not disrupted.

### 2.2. Study Design, Sites, and Population

This retrospective interrupted time series study included patients across ten public primary care clinics within a regional healthcare system serving the eastern and southern regions of Singapore. The study population comprised children aged 6 months to 12 months who attended the well-visits for developmental assessment and vaccinations at the polyclinics. Children in the first cohort were vaccinated according to the pre-intervention schedule (Figure 1) from 14 March 2022 to 14 March 2023, while those in the second cohort were vaccinated according to the visit-aligned schedule (Figure 2) from 15 March 2023 to 15 March 2024. Vaccination records of each study cohort, together with their age, gender, and ethnicity were extracted from the Electronic Medical Records (EMR) database.

The primary outcome of this study was the change in the influenza vaccination rates of children aged between 6 months and 12 months in the two cohorts. This allowed us to evaluate if the scheduling of influenza vaccination to coincide with specific well-visits resulted in higher vaccination rates.

### 2.3. Study Size Determination

The sample size was determined by the total number of eligible children who visited the primary care clinics during the defined observation periods. We conducted a complete enumeration of all children aged 6 to 12 months who attended the primary care clinics during the pre- and post-intervention periods. This inclusion strategy resulted in a final dataset of 19,316 children across both cohorts.

### 2.4. Data Extraction, Processing and Audit

This study analyzed de-identified data extracted from the SingHealth Polyclinics electronic medical records (EMR) system, which captures vaccination records, demographic characteristics, and clinical information across all participating clinics.

Vaccination data for both study cohorts were extracted by research informatics staff from the institutional data repository, including vaccination history, age, gender, and ethnicity. Data were de-identified prior to analysis by an authorized third party to ensure patient confidentiality. Basic data quality checks were performed, including verification of completeness, consistency of vaccination records, and validation of age calculations. The final dataset was transferred securely to the study team for analysis.

### 2.5. Statistical Analysis

Descriptive statistics were reported using frequencies and percentages for categorical variables, while continuous variables were summarized using median and interquartile range. The baseline characteristics between the unmatched pre- and post-intervention groups were compared using an independent *t*-test for continuous variables and the Chi-square test for categorical variables.

An interrupted time series analysis was conducted to address potential sources of bias, particularly time-dependent confounders such as seasonal influenza variations and pre-existing secular trends in vaccine uptake. This approach determined the impact of the intervention by comparing outcome trends before and after the intervention within a single group, without a control group. Additionally, selection bias was reduced by extracting data for the entire eligible population within the institution’s network rather than relying on a sample. Information bias was addressed through the automated extraction of vaccination records from the institutional data repository, independent of recall or self-reporting.

All statistical analyses were performed using Stata MP version 19, with a two-sided significance threshold set at *p* < 0.05.

### 2.6. Ethical Considerations

Ethical review was waived as this study involved analysis of de-identified data in accordance with institutional guidelines.

## 3. Results

### 3.1. Baseline Characteristics of Study Population

The baseline demographic characteristics of the study population (n = 19,316) are summarized in Table 1. The two cohorts had similar median age, gender distribution, and ethnic composition.

### 3.2. Influenza Vaccination Rates in the Study Population

Table 2 shows the influenza vaccination rates and demographic characteristics of the children who received influenza vaccines in the first cohort of 10,082 children and second cohort of 9234 children.

There was a significant difference in the uptake of Dose 1 influenza vaccine between the cohorts. In the first cohort, 4.7% (470 children) received Dose 1 influenza vaccine, compared to 73.7% (6810 children) in the second cohort. The children in the second cohort received Dose 1 influenza vaccine at an earlier median age of 7.6 months compared to 8.7 months in the first cohort (*p* < 0.001).

There was no difference in gender for the uptake of Dose 1 influenza vaccine between the cohorts (*p* = 0.37). There was statistically significant difference in ethnic composition for the uptake of Dose 1 influenza vaccine between the cohorts (*p* < 0.001).

There was a significant difference in the proportion of children who completed two doses of influenza vaccines between the cohorts. In the first cohort, 3.6% (361 children) completed two doses of influenza vaccine, compared to 62% (5729 children) in the second cohort. The children in the second cohort completed their Dose 2 influenza vaccine at an earlier median age of 7.7 months compared to 9.9 months in the first cohort (*p* < 0.001).

There was no difference in gender for the completion of two doses of influenza vaccine between the cohorts (*p* = 0.439). There was statistically significant difference in ethnic composition for the completion of two doses of influenza vaccine between the cohorts (*p* < 0.001).

### 3.3. Longitudinal Impact of Visit-Aligned Schedule on Influenza Vaccine Uptake

**Figure 3:**
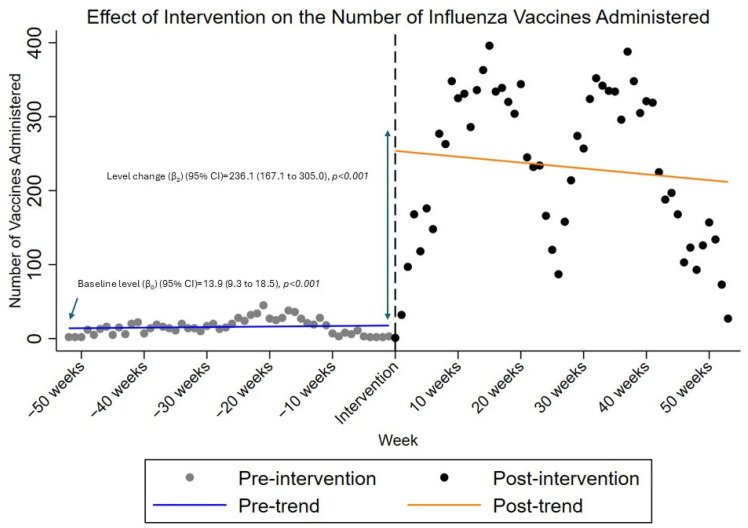
Interrupted time series graph showing the effect of the intervention on the weekly number of influenza vaccine administered. The vertical broken line corresponds to the implementation date (15 March 2023) of the intervention. The grey dots represent weekly influenza vaccines administered before the intervention, while the black dots represent weekly influenza vaccines administered after the intervention. The blue line represents the trend before the intervention, while the orange line represents the trend after the intervention. The green vertical double-headed arrow represents the significant immediate weekly change (β_2_) in influenza vaccines administered after the intervention.

A significant immediate increase was noted in the influenza vaccination uptake rate in children aged between 6 months and 12 months following the implementation of a visit-aligned schedule (Figure 3). Before 15 March 2023, the baseline number of influenza vaccines administered was 13.9 per week (*p* < 0.001). After the implementation on 15 March 2023, there was a significant increase to 236.1 per week (*p* < 0.001), setting a new baseline number of influenza vaccines administered at 250 per week.

The trend was not sustained following the implementation of a visit-aligned schedule (Figure 3 and Table 3). The pre-implementation trend showed a slight increase of 0.073 vaccines per week (*p* = 0.478), indicating relatively stable increase but low vaccine demand over time. The post-implementation trend showed a change of −0.87 vaccines per week compared to the pre-implementation trend (*p* = 0.438), resulting in an overall post-implementation slope of −0.80 vaccines per week.

## 4. Discussion

This study demonstrates that implementing a visit-aligned schedule from a flexible, discretionary model to a defined visit-aligned schedule significantly improved influenza vaccine uptake among young children in a primary care setting. Dose 1 vaccination rates increased dramatically from 4.7% to 73.7%, and two-dose completion rates rose from 3.6% to 62% following the intervention. These findings suggest that alignment of vaccination schedules with existing clinical touchpoints is a highly effective strategy for addressing low influenza vaccine uptake in this age group.

The substantial increase in uptake rates observed in this study is consistent with broader evidence that integrating preventive health interventions into routine care reduces missed opportunities for vaccination [22,23]. By embedding the influenza vaccine into the 6-month and 7-month touchpoints, we removed the ambiguity of when to vaccinate, changing the default status from “opt-in” to “routine.” This aligns with behavioral science principles where default options significantly influence health decisions [24]. The creation of a dedicated 7-month visit for the Dose 2 influenza vaccine ensured that the completion of the primary series was not left to chance or ad hoc visits, directly addressing the barrier of multiple injections per visit by spacing them out appropriately.

The reduction in median age at first dose (from 8.7 to 7.6 months) and at two-dose completion (from 9.9 to 7.7 months) is also clinically meaningful. Earlier vaccination reduces the window of vulnerability to influenza-related complications, which are disproportionately severe in infants and young children [1,2,3,4,5,6]. This aligns with recommendations from health authorities that children in this age group be vaccinated as early as eligible [8].

Implementing this schedule required consideration of the burden of multiple injections and potential stakeholder resistance. Although there is no defined upper limit to the number of injectable vaccines that can be safely administered in a single visit [9], studies have shown that both caregivers and healthcare providers are more comfortable with one to two injections per visit [14,16]. Concerns regarding multiple injections are consistently reported and include perceived pain, risk of adverse events, and the overall burden on the child. Importantly, provider perceptions of parental concern may influence clinical practice, with evidence suggesting that providers may defer recommended vaccinations when they anticipate resistance to multiple injections, potentially leading to delays or incomplete immunization [15]. To maintain a limit of two injections per visit which is a key concern for parents and providers while preserving adherence to national immunization recommendations, Dose 2 of the PCV13 vaccine was rescheduled to the new 7-month touchpoint. This decision was intended to avoid any potential increase in post-vaccination febrile admissions associated with shifting the dose to an earlier age [25,26]. This was supported by local epidemiological data showing a lower burden of invasive pneumococcal disease in this age group [21].

Despite these encouraging results, several limitations should be acknowledged. As a single-healthcare-cluster study, the generalizability of findings to other settings with different primary care models may vary. However, this study included data from ten polyclinics within the cluster, serving a large and diverse population. The care model and workflow are comparable across public primary care institutions in Singapore, which supports the broader applicability of our findings.

Retrospective electronic medical records data extraction may miss vaccinations administered outside our network. Furthermore, data regarding specific reasons for non-uptake, such as parental refusal, vaccine hesitancy, or acute medical deferrals, were not captured in our dataset. In addition, acceptability among healthcare providers and parents was not formally assessed using structured instruments. However, the successful implementation across multiple clinics without disruption to routine workflows suggests good operational feasibility and general acceptance. The unavailability of these qualitative data limits our ability to fully account for the factors contributing to the remaining proportion of children who did not receive the vaccine despite the optimized schedule.

Although a contingency protocol was implemented to manage vaccine supply interruptions, its activation frequency and effectiveness were not systematically captured and therefore could not be evaluated.

The long-term cost-effectiveness of this model in terms of manpower utilization and clinic time was not evaluated. However, the substantial improvement in vaccination uptake and earlier completion of the primary series suggests potential downstream benefits, including reduced influenza-related healthcare utilization. Future studies should evaluate the economic and operational impact of this approach, including its effects on consultation time, staffing requirements, and cost per fully vaccinated child, explore parental and provider perspectives, identify residual barriers, evaluate the long-term impact on reducing influenza-related pediatric hospitalizations and morbidity and assess the scalability of this model across diverse healthcare settings.

## 5. Conclusions

Aligning childhood influenza vaccination with specific well-visits proved to be a highly effective strategy for improving immunization coverage in a primary care setting. By operationalizing the national guidelines into a fixed 6-month and 7-month workflow, we successfully normalized the delivery of the influenza vaccine, resulting in a 17-fold increase in uptake and significantly earlier completion of influenza vaccination in young children. These findings have meaningful implications and future research potential for preventive care delivery and public health policy.

## Figures and Tables

**Table 1:** Baseline characteristics of study population.

	First Cohort	Second Cohort	*p*-Value
Total number of children, n	10,082	9234	
Median age, in months (IQR)	6.9 (6.6–7.3)	6.9 (6.6–7.3)	0.002
Gender			0.119
Male, n (%)	5294 (52.5)	4745 (51.4)	
Female, n (%)	4788 (47.5)	4489 (48.6)	
Ethnicity			0.971
Chinese, n (%)	6041 (59.9)	5531 (59.9)	
Malay, n (%)	2640 (26.2)	2427 (26.3)	
Indian, n (%)	828 (8.2)	765 (8.3)	
Others, n (%)	573 (5.7)	511 (5.5)	

**Table 2:** Influenza vaccination rates and characteristics of children who received influenza vaccines.

	Dose 1 Influenza Vaccine			Dose 2 Influenza Vaccine		
	First Cohort n = 10,082	Second Cohort n = 9234	*p*-Value	First Cohort n = 10,082	Second Cohort n = 9234	*p*-Value
	470 (4.7)	6810 (73.7)		361 (3.6)	5729 (62.0)	
Median age, in months (IQR)	8.7 (8.0–9.7)	7.6 (6.9–8.3)	<0.001	9.9 (9.1–10.8)	7.7 (6.9–8.4)	<0.001
Gender			0.37			0.439
Male, n (%)	232 (4.4)	3507 (73.9)		178 (3.4)	2945 (62.1)	
Female, n (%)	238 (5.0)	3303 (73.6)		183 (3.8)	2784 (62.0)	
Ethnicity			<0.001			<0.001
Chinese, n (%)	350 (5.8)	4145 (74.9)		272 (4.5)	3555 (64.3)	
Malay, n (%)	60 (2.3)	1842 (75.9)		42 (1.6)	1443 (59.5)	
Indian, n (%)	34 (4.1)	564 (73.7)		27 (3.3)	440 (57.5)	
Others, n (%)	26 (4.5)	379 (74.2)		20 (3.5)	311 (60.9)	

**Table 3:** Segmented regression coefficients from interrupted time series analysis.

Parameter	Description	Coefficient	*p*-Value	95% CI
Baseline level (β_0_)	Average weekly vaccines before intervention	13.93469	<0.001	9.323387 to 18.54599
Pre-intervention trend (β_1_)	Weekly change before intervention	0.072697	0.478	−0.1280411 to 0.2734351
Level change (β_2_)	Immediate change after intervention	236.1026	<0.001	167.1994 to 305.0057
Trend change (β_3_)	Change in weekly trend after intervention	−0.86595	0.438	−3.051982 to 1.320081

## Data Availability

Data are contained within the article.

## Abbreviations

The following abbreviations are used in this manuscript:

NCIS	National Childhood Immunization Schedule
LRTI	Lower respiratory tract infection
WHO	World Health Organization
BCG	Bacillus Calmette–Guérin
HepB	Hepatitis B
DTaP	Diphtheria, tetanus and acellular pertussis
IPV	Injectable polio vaccine
MMR	measles, mumps and rubella
MMRV	measles, mumps, rubella and varicella
PCV13	13-valent pneumococcal conjugate vaccine
IPD	Invasive pneumococcal disease
SHP	SingHealth Polyclinics
EMR	Electronic Medical Records
